# EXTENSIVE CONGENITAL VULVAR LYMPHANGIOMA MIMICKING GENITAL WARTS

**DOI:** 10.4103/0019-5154.60372

**Published:** 2010

**Authors:** Vandana Mehta, Sudhir Nayak, C. Balachandran, Puja Monga, Raghavendra Rao

**Affiliations:** *From the Department of Skin and STD, Kasturba Medical College, Manipal, Karnataka, Dr. Vandana Mehta, Department of Skin and STD, Kasturba Medical College, Manipal - 576 104, Karnataka, India. E-mail: vandanamht@yahoo.com*

Sir,

Lymphatic malformation (LM) or lymphangioma is a benign proliferation of the lymphatics accounting for four per cent of all vascular malformations and 26% of all benign vascular tumors.[1,2]

**Figure 1:**
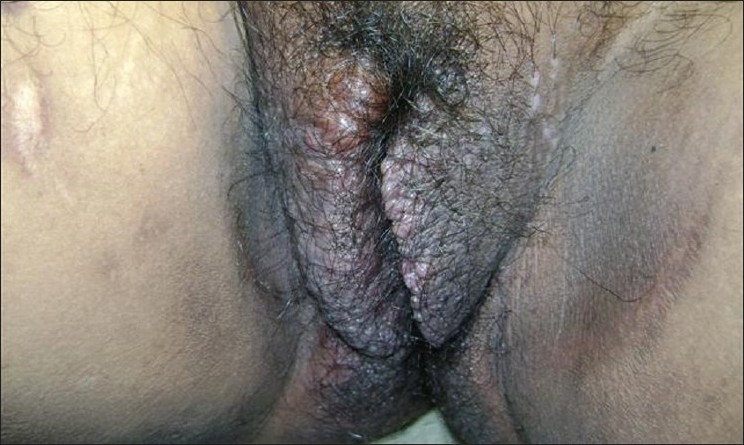
Clinical picture shows vulvar hypertrophy with a rugose appearance

**Figure 2:**
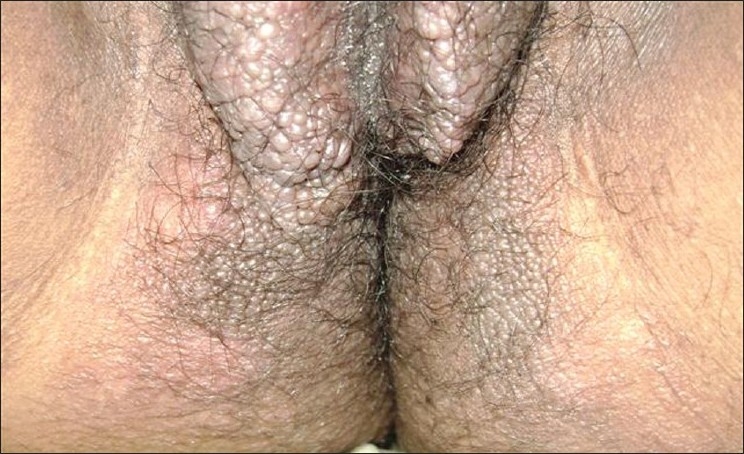
Close up shows multiple superficial skin colored shiny papules and warty excrescences

A 26-year-old female presented with vulvar hypertrophy since 10 years of age, which gradually increased in size after puberty. It was associated with episodic pain on and off interfering with walking. There was no history of oozing. Cutaneous examination of the external genitalia revealed gross vulvar hypertrophy extending upto the fourchette. The overlying skin was hyper- pigmented, indurated, rugose and studded with multiple skin colored papules. [Figures 1 and 2] Based on the clinical appearance, a differential diagnosis of vulvar lymphangioma and genital warts was entertained. Biopsy for histopathology revealed dilated lymphatics lined with flattened endothelial cells and luminal macrophages, neutrophils and lymphocytes consistent with our diagnosis of lymphangioma.

LMs are broadly classified into superficial lymphangioma circumscriptum and deeper cavernous lymphangioma.[3] There is no clear distinction between both, the difference being solely on the extent of the malformation.[4] Differentiation between congenital vs. acquired lymphangiomas with respect to localization within the skin have been made. The former result from a hamartomatous malformation of lymphatic vessels, the latter from acquired obstruction of lymph vessels, e.g. after surgical or radiation treatment of malignancies of the breast or uterus.[5] Acquired lymphangioma of the vulva, arising without obvious causes, seems to be unusual.[6]

Lymphangiomas of the vulva are rare. Thirteen cases of the congenital and 24 cases of acquired form have been reported in the literature presently.[7] Diffuse lymphangiomas, though present from birth, may go unnoticed for many years. They present as asymptomatic, erythematous flat indurated or atrophic plaques. Swelling may or may not be apparent. Diffuse lymphangiomas, unlike the superficial type, may not have any surface changes. The diagnosis is usually made by biopsy.[7]

Various modalities of treatment have been suggested viz., observation, surgical excision of skin and subcutaneous tissues, surface ablation with Laser (CO_2_, Er:YAG), sclerotherapy, superficial radiotherapy.[8–10] Our patient was advised vulvectomy but she was lost for follow-up.
